# Psychological Aspects and Eating Habits during COVID-19 Home Confinement: Results of EHLC-COVID-19 Italian Online Survey

**DOI:** 10.3390/nu12072152

**Published:** 2020-07-19

**Authors:** Laura Di Renzo, Paola Gualtieri, Giulia Cinelli, Giulia Bigioni, Laura Soldati, Alda Attinà, Francesca Fabiola Bianco, Giovanna Caparello, Vanessa Camodeca, Elena Carrano, Simona Ferraro, Silvia Giannattasio, Claudia Leggeri, Tiziana Rampello, Laura Lo Presti, Maria Grazia Tarsitano, Antonino De Lorenzo

**Affiliations:** 1 Section of Clinical Nutrition and Nutrigenomic, Department of Biomedicine and Prevention, University of Tor Vergata, Via Montpellier 1, 00133 Rome, Italy; laura.di.renzo@uniroma2.it (L.D.R.); paola.gualtieri@uniroma2.it (P.G.); delorenzo@uniroma2.it (A.D.L.);; 2 School of Specialization in Food Sciences, University of Rome Tor Vergata, Via Montpellier 1, 00133 Rome, Italy; alda.attina@gmail.com (A.A.); kikafabiola@gmail.com (F.F.B.); caparello.giovanna@gmail.com (G.C.); vanessacamodeca@libero.it (V.C.); elena_carrano@libero.it (E.C.); ferrarosimona@hotmail.it (S.F.); silviagiannattasio85@gmail.com (S.G.); claudialeggeri@gmail.com (C.L.); tizianarampello1@gmail.com (T.R.); 3 Predictive and Preventive Medicine Research Unit, Bambino Gesù Children’s Hospital IRCCS, 00165 Rome, Italy; 4 Department of Physic, University of Rome Sapienza, P.zza Aldo Moro 5, 00185 Rome, Italy; bigionigiulia@gmail.com; 5 Department of Health Sciences, University of Milan, Via A. Di Rudinì, 8, 20142 Milan, Italy; laura.soldati@unimi.it; 6 Unitelma Sapienza, University of Rome Sapienza, Via Regina Elena, 295, 00161 Rome, Italy; la.lopre@gmail.com; 7 Department of Experimental Medicine, University of Rome Sapienza, Rome 00161, Italy; mariagrazia.tarsitano@uniroma1.it

**Keywords:** SarsCoV2, COVID-19, lockdown, psychological effects, emotional eating, lifestyle, eating behaviours

## Abstract

The COVID-19 pandemic has had a huge impact on the population with consequences on lifestyles. The aim of the study was to analyse the relationship between eating habits, mental and emotional mood. A survey was conducted online during social isolation, from 24 April to 18 May 2020, among the Italian population. A total of 602 interviewees were included in the data analysis. A high percentage of respondents experienced a depressed mood, anxious feelings, hypochondria and insomnia (61.3%, 70.4%, 46.2% and 52.2%). Almost half of the respondents felt anxious due to the fact of their eating habits, consumed comfort food and were inclined to increase food intake to feel better. Age was inversely related to dietary control (OR = 0.971, *p* = 0.005). Females were more anxious and disposed to comfort food than males (*p* < 0.001; *p* < 0.001). A strength of our study was represented by the fact that the survey was conducted quickly during the most critical period of the Italian epidemic lockdown. As the COVID-19 pandemic is still ongoing, our data need to be confirmed and investigated in the future with larger population studies.

## 1. Introduction

The new form of coronavirus (Sars-CoV-2) has triggered a worldwide state of emergency [1]. In this pandemic scenario, the experts involved [2] are increasingly concerned by the psychological implications that the epidemic has brought with it, especially for elderly people with compromised immune systems and for the health of the workers employed on the front lines against this virus [3]. Previous studies have revealed a wide range of psychosocial impacts on individuals and on the overall community during outbreaks of infections [4]. On a personal level, people experienced fear of getting sick or dying, feelings of helplessness and stigma [4]. In particular, the fear of one’s own health and of their loved ones, social distancing and the quarantine obligations have put a strain on the affective and emotional sphere of every individual. This situation has severely undermined the psychological stability of Italians as well as the worldwide population, causing adverse psychological effects.

The lockdown measures have had a great impact on everyday life [2], often associated with a negative influence on psychological well-being. These circumstances have exasperated a series of psychological and psychopathological conditions, including emotional exhaustion, irritability, anxiety, increased anger, depressive symptoms as well as a post-traumatic stress disorder [5]. Psychological theories, such as the behaviour immune system (BIS), argue that these emotional and cognitive responses support proactively the immune system in the fight against the pathogen agents [6].

Until now, the information on psychological impact of the COVID-19 pandemic on the population continues to be limited. Researchers, in fact, have mainly focused on identifying epidemiology and clinical features of infected patients [7], virus genomics characterizations [8] and governmental challenges in the healthcare and economic fields [9]. Besides these priorities, it is important not to downplay the contribution of social and behavioural sciences in shaping and optimizing individual and collective response to the crisis [10]. During an epidemic, people can react to official information in an irrational way and, thus, governments should make people aware of the situation without raising alarms [11].

Several studies have highlighted how a significant number of individuals have manifested a series of psychological effects and the difficulties in adapting to the new lifestyle of the quarantine. In China, the psychological impact and the state of mental health during the first phase of COVID-19 pandemic were assessed through the Event Scale (IES-R), while the impact of depression, anxiety and stress were assessed by the Depression Anxiety and Stress Scale (DASS-21) [12]. Similarly, an Italian study assessed the general population’s psychological distress during the pandemic through an online survey. This showed that 38% of the population was affected by significant psychological indispositions [13]. Further, many studies have compared the psychological outcomes between quarantined and non-quarantined people [14]. One in particular demonstrated that the quarantine effect could be a predictor of post-traumatic stress symptoms, even years after the event [15]. In Poland, a significant percentage of individuals changed dietary habits and started eating and snacking more, leading to weight gain in overweight and obese subjects [16]. Therefore, as widely demonstrated by these studies, negative effects of post-traumatic stress symptoms, confusion and anger were reported. The stress factors included uncertainty about the duration of the quarantine, fear of possible infections, the ban on going to hospitals unless strictly necessary, frustration, boredom, infodemic, overall uncertainty of the future, fear of significant financial losses and long-term repercussions that the country will face.

It is pivotal to highlight how containment measures, including self-isolation and social distancing, may have had a strong impact on the everyday life of the population and how the population’s psychological well-being may have been negatively affected.

The “Eating Habits and Lifestyle Changes in COVID-19 lockdown” (EHLC-COVID19) project on the Italian population has started to explore and analyse, in a diachronic perspective, the multi-dimensional lifestyle behaviours, eating habits and mental and emotional responses during home confinement [16].

The first survey launched with the EHLC-COVID19 and focused on eating habits, adherence to the Mediterranean Diet (MD) and the changes in the lifestyle faced during the COVID-19 lockdown of the Italian population [17]. This paper presents data from the second part of the EHLC-COVID19 survey that aimed to analyse the psychological status during the COVID-19 pandemic and its correlation with the eating habits in the Italian population.

## 2. Materials and Methods

### 2.1. Survey Methodology and Promotion

The EHLC-COVID19 project conducted research, using an electronic survey in Italian, to collect data on the Italian population regarding eating habits, lifestyle and the behavioural and emotional impact related to the COVID-19 pandemic.

The survey was designed by a steering group of scientists at the Section of Clinical Nutrition and Nutrigenomics at the Department of Biomedicine and Prevention of the University of Rome Tor Vergata. It was conducted during the lockdown period among the Italian population using an online platform (Google Form) which was accessible by any device with an Internet connection. The survey was concluded when the Italian ministerial ordinances started authorising again some public and private activities. The questionnaire was uploaded and shared through the institutional mailing list, social networks (Twitter, Instagram and Facebook), and the “PATTO in Cucina Magazine” website [17]. The online survey provided statistical collective parameters. The research objectives were entirely successful, since this method facilitated the wide dissemination of the questionnaire without any type of limit.

According to the latest Italian Annual Report on Internet Access, the selected methodology conformed with the actual use of the Internet in Italy. In January 2020, 94% of internet visitors, aged 16 to 64, used their mobile phones to navigate the Web, while 99% of them used specifically social networks and messaging services [18].

The survey included an introductory page describing the background, the aims and information on the ethics of the survey. The inclusion criteria for the respondents were: people living in Italy, age 18–79 years, female or male. Individuals living outside of Italy were excluded. These criteria were verified by answers given to the corresponding survey questions.

The structured questionnaire included 25 questions, divided into three different sections: (1) personal and general data (including 6 questions: age, gender, information on region and province of residence, level of education, and cohabitation situation at home); (2) anthropometrics information (including 2 questions reported as weight and height); (3) lifestyle, eating habits changes, psychological and emotional aspects caused by the social isolation during the pandemic period (including 17 questions modified from validated tools [19,20,21,22,23]), to investigate and assess the emotional aspects such as anxiety, depressed mood, hypochondria, level of concern, emotional eating, insomnia, dietary changes, as well as the perception of diet control and appetite. No names or other personal information was requested.

The questions within the last section of the survey were extrapolated from the 14 item Hamilton Anxiety Rating Scale [19,20], commonly used in the clinical context to evaluate anxiety symptoms, the 17 item Hamilton Depression Scale [21], developed to assess depression and from the 25 item Yale Food Addiction Scale (YFAS), designed to identify those exhibiting signs of addiction towards certain types of foods [22,23]. Only some of the questions, from the Italian version of the scales, were used and edited by researchers to adapt them to the current period of social isolation, hence, no scoring scale was calculated. The full version of the questionnaire, translated into English, is available in Appendix A.

The online survey was conducted in full agreement with the national and international regulations in compliance with the Declaration of Helsinki (2000). All participants were fully informed about the study requirements and were required to accept the data sharing and privacy policy before taking part in the study. To maintain and protect the confidentiality of the participants, their personal information and data were anonymous, according to the provisions of the General Data Protection Regulation (GDPR 679/2016). The anonymous nature of the web survey did not allow for tracing in any way sensitive personal data. Therefore, the present web survey study did not require approval by the Ethics Committee.

The participants completed the questionnaire directly connected to the Google Form, each questionnaire was sent to the final database and downloaded as a Microsoft Excel sheet. The participants’ answers were anonymous and confidential according to Google’s privacy policy [24]. The participants would have been able to withdraw their participation in the survey at any stage before the submission; non-completed responses were not saved.

### 2.2. Statistical Analysis

Descriptive statistics were employed to explore demographic, personal characteristics and anthropometric parameters of the study sample. Data are represented as numbers and percentages in parentheses (%) for categorical variables or mean and standard deviation (SD), as well as median and interquartile range in square brackets [IQR] for continuous variables. The Shapiro–Wilk test was carried out to evaluate variables distribution. All the variables had non-normal distribution. The Spearman correlation coefficient was calculated to evaluate the correlation between continuous variables. The Chi-square test was employed to assess the association of categorical variables. Instead, Mann–Whitney U and Kruskal–Wallis tests were performed to compare continuous variables among two or more groups, respectively. Finally, univariable and multivariable binary logistic regression analyses were conducted to investigate the association between categorical variables (dependent) and continuous or categorical ones (independent). Results were significant for *p*-value < 0.05. Statistical analysis was performed using SPSS ver. 21.0 (IBM, Chicago, IL, USA).

## 3. Results

### 3.1. Participants

The web survey was launched on the 24 April 2020 and concluded on the 18 May 2020, when the lockdown in Italy ended (Appendix B shows the geographical distribution of COVID-19 total positive cases in Italy on 18 May 2020). Thereafter data were analysed. A total of 700 participants completed the questionnaire. After the validation of the data, 602 respondents, aged between 18 and 79 years, were included in the analysis. The female respondents represented the majority of the population (79.7%). The territorial coverage spread over all Italian regions: 15.6% of respondents lived in Northern Italy, 40.0% in Centre Italy, and 44.4% in Southern Italy and Islands. According to the age distribution, the sample reflected the population of Italian internet users (i.e., 98.7% of people older than 20 years) [25]. General characteristics and anthropometrics of the population are reported in Table 1. A positive correlation between BMI and age was found (*r* = 0.296, *p* < 0.001). No difference was found in BMI when comparing the different Italian regions (*p* = 0.078), while males showed a significantly higher BMI in comparison to females (Mann–Whitney U = 20,331.50, *p* < 0.001).

For what concerns cohabitation during the COVID-19 emergency, 192 (31.9%) participants declared to live with their parents, 157 (26.1%) with their partner and children, 134 (22.3%) with just their partner and 21 (3.5%) with just their children. Finally, 64 (10.6%) of them declared to live alone and 34 (5.6%) with flatmates. Furthermore, 196 (31.9%) of the respondents affirmed to suffer from a disease (e.g., hypertension, oncological, cardiovascular or autoimmune diseases).

### 3.2. Emotional State during the COVID-19 Emergency

**Figure 1:**
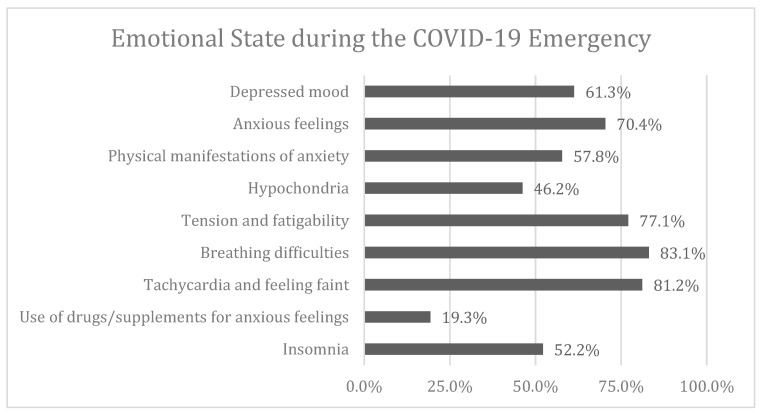
Percentage of positive answers to the questions extrapolated from the Hamilton Depression Rating Scale about depressed mood, anxious feelings, the physical manifestations of anxiety (tachycardia, headache, sweating), hypochondria, tension and fatigability (on alert, ready to cry, trembling, restless, unable to relax), breathing difficulties (sighing, choking sensation, chest pressure, dyspnoea), tachycardia and feeling faint (palpitation, chest pain), use of drugs and supplementation for anxious mood. The figure also includes the percentage of positive answers about insomnia.

With regards to the emotional state, a high percentage of the respondents declared to have felt anxious and depressed during the COVID-19 lockdown. Figure 1 shows the percentage of positive answers to the questions extrapolated from the Hamilton Depression Rating Scale. The figure includes also the percentage of positive answers concerning insomnia.

Considering the different Italian regions, a difference was found for physical manifestation of anxiety (*p* = 0.046; percentage of positive answers: North 55.3%, Centre 52.7%, South and Islands 63.3%) and tension (*p* = 0.017; percentage of positive answers: North 84.0%, Centre 71.4%, South and Islands 79.8%). Moreover, a difference among age groups was found for depressed mood, anxious feelings and insomnia (*p* = 0.001, *p* < 0.001 and *p* = 0.014, respectively). In particular, the univariable binary logistic regression showed that age was inversely correlated to these emotional states (depressed mood: OR = 0.980, *p* = 0.002; anxious feelings: OR = 0.966, *p* < 0.001; insomnia: OR = 0.980, *p* = 0.001).

**Figure 2:**
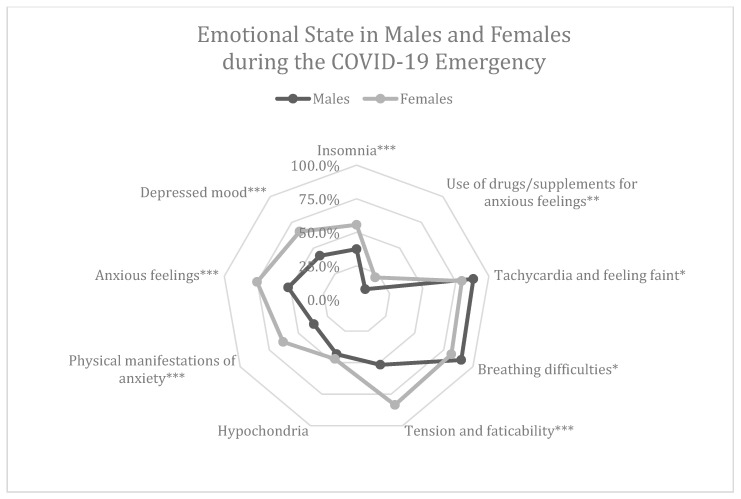
Percentages of positive answers to questions related to the emotional state during the COVID-19 emergency, in males and females. A Chi-square analysis was performed to compare male and female percentages. * *p* < 0.05; ** *p* < 0.01; *** *p* < 0.001.

With regards to gender, the percentage of females declaring to feel depressed and anxious, to experience physical manifestations of anxiety, tension and insomnia problems during the COVID-19 emergency was significantly higher than the males one (at the *Chi*-Square analysis *p* < 0.001 for all the variables). Females were also more prone to take drugs or supplements for their anxious feelings (*p* = 0.006). Surprisingly, a higher percentage of males in comparison to females affirmed to have felt breathing difficulties and other symptoms such as tachycardia or perception of fainting (*p* = 0.028; *p* = 0.035). No difference between males and females was found for hypochondria (*p* = 0.475). Results are shown in Figure 2. Further, 24.1% of the respondents declared to have stopped working during the pandemic, while 36.9% affirmed to have had difficulties in concentration in their daily work.

### 3.3. Emotional Eating Behaviour during the COVID-19 Emergency

**Figure 3:**
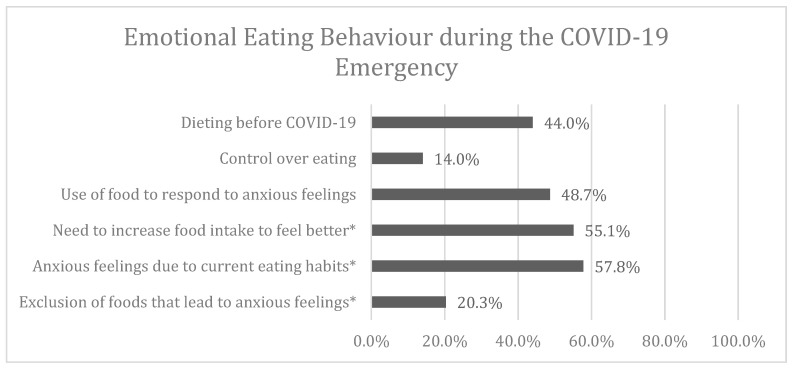
Percentage of positive answers to the questions about emotional eating behaviour. * Questions extrapolated from the Yale Food Addiction Scale.

With regards to the emotional eating behaviour during the COVID-19 isolation, almost half of the respondents declared to have felt anxious due to the fact of their eating habits. They admitted to having used food as a means of comfort in response to their anxious feelings and to being prone to increasing their food intake to feel better. Figure 3 shows the percentage of positive answers to the questions concerning emotional eating behaviour, including those extrapolated from the Yale Food Addiction Scale.

No difference was found for the emotional eating behaviour in the different Italian regions. In the regression analysis no correlation was found between age and the different emotional eating behaviour (need to increase food intake: *p* = 0.441; use of food to respond to anxious feelings: *p* = 0.441; anxious feelings due to the eating habits: *p* = 0.327; foods exclusion: *p* = 0.454; dieting before COVID-19: *p* = 0.495). On the contrary, age resulted to be inversely correlated to the control overfeeding (OR =0.971, *p* = 0.005).

**Figure 4:**
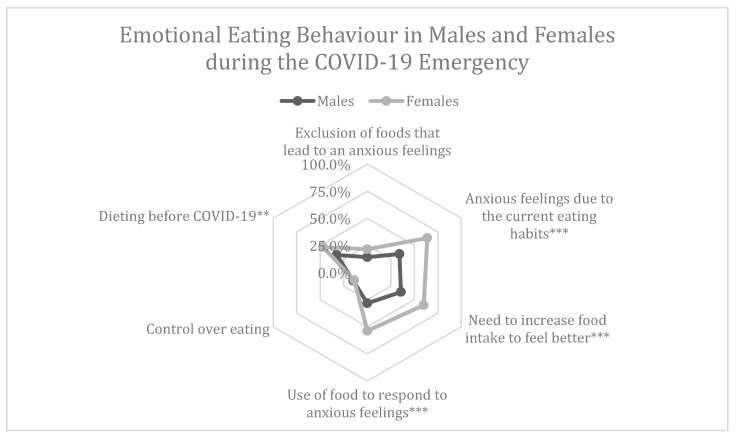
Percentages of positive answers to questions related to the emotional eating behaviour during the COVID-19 emergency in males and females. A Chi-square analysis was performed to compare male and female percentages. ** *p* < 0.01; *** *p* < 0.001.

With regards to gender, in comparison to males, a higher percentage of females was on a diet before the COVID-19 emergency (*p* = 0.005). Moreover, females declared to be more prone to emotional eating, needing to increase their food intake to feel better or using food as a response to their anxious state (*p* < 0.001; *p* < 0.001). Finally, due to the fact of their eating habits, they also felt more anxious when compared to males during the COVID-19 lockdown (*p* < 0.001). No difference was found for the control of over-eating (*p* = 0769) and exclusion of foods that lead to anxious feelings (*p* = 0.096). Results are shown in Figure 4.

### 3.4. Eating Control and Emotional State

The multivariable binary logistic regression analysis was performed to evaluate which factors could have been predictors of the ability to control over-eating during the pandemic. The results of the univariable analysis are shown in Table S1. The final step of the backward approach is shown in Table 2. The increased control of over-eating during the lockdown was associated with lower age, lower BMI, not feeling anxious, dieting before COVID-19 and being less prone to increase food intake to feel better.

## 4. Discussion

There are different studies and surveys created all over the world that demonstrate that the COVID-19 lockdown has affected the population’s psychological wellness [26,27,28]. The choice of quarantine from public health institutions has generated positive effects on the hindrance of the spread of the virus but contemporarily led to many symptoms of emotional uncomfortableness and psychological disorders in the population [29]. The severe quarantine restrictions, such as social distancing, school and several work activities closing, the ban on group gatherings and physical activities in open spaces and dedicated facilities, abruptly turned upside down the traditional lifestyle. It generated consequences on the psychological and emotional state globally [2]. The second part of the EHLC-COVID19 project [18] started in this period of social constrain to evaluate consequences on mood and nutrition habits of 602 individuals.

The respondents to the questionnaire were mainly females from the different Italian regions, young individuals and a large portion of them cohabiting within their family. The lockdown has undoubtedly had effects on the mood of the participants of the survey: 61.3% of the respondents said that they have had, for various reasons, a lowering of their mood. The majority of the participants in the survey referred to anxious feelings and depressed moods as well as exhaustion and tension with tachycardia and breath difficulties. The low mood was not directly connected to a clinical diagnosis of a depressive state but to an emotional state. Nonetheless, the symptoms of depression, besides the evaluation of the mood tone, were also connected to behavioural and cognitive evaluations (hypersomnia/hyposomnia, hyperphagia/hypophagia, lack of concentration, attention, etc.) [30]. Moreover, 36.9% of the interviewees claimed to have reduced their concentration in their working activity. The majority of respondents (70.4%) reported having experienced anxious feelings, yet it is unsure whether this state was pathological or simply related to the lockdown. Anxiety is a natural emotional state that causes people to perceive themselves to be in danger when they can no longer manage to implement their forecasting system [31]. In the time of the COVID-19, anxiety can be considered a natural consequence and not necessarily an indicator of endogenous disturbance, it is rather reactive and connected to the perceived danger. The anxious symptomatology, where present, was expressed with mild or moderate symptoms and nobody claimed to have had crippling/disabling experiences; when present, rather than appearing with specific physical symptoms and in a somatised form (tachycardia, tremor, sweating, etc.) it seemed to express itself as an inability to relax and as a state of nervousness and restlessness. On the other hand, 46.2% had health concerns and a fear of get sick. In almost all the interviewees, the need to use specific drugs or supplements for the management of anxiety was not reported. This may be a result of the low intensity of the symptoms as well as the interpretation of this state as a normal consequence of the situation experienced, not only by the specific subject but by the overall population. This underlies the belief that, where “collective” emotional states and situations of shared danger are experienced, there is the perception of being in the “norm”. As such, individuals feel like they belong to a group which therefore makes them feel less isolated and capable of being able to count on the protection from others.

However, with regards to the gender, we discovered that the pandemic has caused, in females, a depressive mood, anxious state, the manifestation of anxiety, tension and insomnia; their use of drugs and supplements increased significantly contrary to that of males, who instead suffered from psychosomatic effects like tachycardia and breath difficulties. This was probably because the anxious individual has a higher physiological response to stressful stimuli and is more frequent in the female gender [32]. In addition, the results have shown an inverse correlation between the age of respondents and the presence of depressed mood, anxious feelings and insomnia.

The survey also investigated the relationship between the psychological state and emotional eating. Emotional eating refers to the drive to eat as a reaction to negative feelings or stress. Negative emotions like anxiety, stress and depression could be a leading cause for the insurgency of emotional hunger [33,34]. Almost a half of the respondents (44.0%) followed a dietary diet, before the outbreak of the pandemic, highlighting a natural predisposition to “dieting” by the female population. The lockdown seems to have influenced the ability to control the relationship with food. Isolation, lack of stimuli, boredom and changing food routines had effects on 86.0% of respondents who reported that they were unable to sufficiently control their diet. We could suppose that there was a variation of caloric intake of each meal due to the quantity and quality of food daily consumed in the quarantine period, and a major number of highly elaborated homemade foods and of superior caloric content [17,35]. We know that there are no foods or natural remedies that can prevent COVID-19 infections [36]; nevertheless, an anti-inflammatory diet could be useful to strengthen the immune system and contrast inflammatory cascade and oxidative stress [37]. Butler et al. [38] suggested that the type of diet can influence both the host’s response and the pathogen’s virulence. In particular, there could be a correlation between the consumption of high palatable foods, like ultra-processed ones, and an impairment of the temporal coordination of the innate and adaptive immunity. Such impairment has been shown to increase the probability of infection by COVID-19, as well as of a more severe clinical course.

The enhanced exposure to food caused by the increase of boredom and having more time available to cook and consume the meal, also enhanced by the fact that the only freedom allowed was to go grocery shopping, induced people who least succeed in managing their diet to amplify the relationship between food intake and emotions. Despite this awareness, “containment” actions have not been put into practice. Many individuals have chosen not to limit themselves, except on rare occasions.

It emerges also that there is a difference in gender regarding emotional hunger. Females display a higher state of eating anxiety compared to males. The results show that females had more alimentation anxiety and felt the need to increase food intake in comparison to males. This is probably caused by the female physiology which is more subject to emotional hunger and to symptoms of depression [39]. We could assess a correlation between anxiety, depressive mood and food dependency which could lead to a food addiction, referring to the idea that in some sensible subjects some highly palatable edibles foods would generate a process comparable to addiction [40]. More specifically the definition of this condition is complex and highly debated: it encompasses emotional, behavioural, cognitive and physiological aspects [41]. Consumption of palatable food can have positive and strengthening effects. It can sensibly normalise stress response with the optimizing and comforting effects [42]. Specific nourishments, mainly those rich in fats and/or sugars, may induce behaviour similar to “addiction” and, in certain conditions, generate neuronal changes. These consumption models are associated to enhanced risks of comorbidity conditions as obesity, early weight gain, depression, anxiety, substance abuse as well as relapse and treatment problems [43].

On the one hand, the lockdown has allowed more room for imagination and exploration with food both in terms of recipes and human relations (for example cooking and eating together more often than before), on the other hand, some individuals have experienced an increase of boredom, general inactivity and seeking out new stimuli in food.

Lastly, by analysing different variables that include age, BMI and anxiety mood, it was possible to observe that during the quarantine the younger population with lower BMI had suffered less the increase in food control and decrease of food intake. This should be further investigated with deeper studies and among a larger sample of people. It should take into consideration whether there are differences among the different Italian regions, as the COVID-19 infection has had a diverse spread between Northern, Central and Southern Italy.

From a psychological point of view, resilience is the ability to face and overcome a dramatic event or a difficult period. The lockdown caused by the COVID-19 pandemic has heavily influenced our life by completely changing our routines and isolating us from our loved ones. Italians have demonstrated courage and strong resilience to maintain a normal lifestyle and discreet eating habits, even when sanitary and economic situations were hard to handle.

A strength of our study was represented by the fact that the survey was conducted quickly in the most critical period of the lockdown in Italy. As the COVID-19 pandemic is still ongoing, our data need to be confirmed and investigated in the future with larger population studies. The main limitations of this study are related to the lack of test scoring and of some data we could have collected which may have increased the psychological strain, such as COVID-19 diagnosis and economic status. Hence, further study on psychological status, eating habits and positivity in relation to COVID-19 should be conducted.

## Figures and Tables

**Table 1:** Population’s characteristics and anthropometrics.

	Whole Sample (*n* = 602)	Northern Italy (*n* = 94)	Centre Italy (*n* = 241)	Southern Italy and Islands (*n* = 267)
Age	36.0 [20.0]	36.0 [18.0]	33.0 [23.0]	38.0 [17.0]
	38.2 ± 12.9	38.1 ± 12.5	37.3 ± 14.1	39.1 ± 11.9
**Age Groups**				
18–30 years	212 (35.2%)	33 (35.1%)	107 (44.4%)	72 (27.0%)
31–50 years	279 (46.3%)	45 (47.9%)	86 (35.7%)	148 (55.4%)
51–65 years	91 (15.1%)	13 (13.8%)	37 (15.4%)	41 (15.4%)
>66 years	20 (3.3%)	3 (3.2%)	11 (4.6%)	6 (2.2%)
**Gender**				
Female	480 (79.7%)	82 (87.2%)	182 (75.5%)	216 (80.9%)
Male	120 (19.9%)	12 (12.8%)	58 (24.1%)	50 (18.5%)
Not specified	2 (0.3%)	0.0 (0%)	1 (0.4%)	1 (0.4%)
**Educational Level**				
Compulsory school	44 (7.3%)	9 (9.6%)	11 (4.6%)	24 (9.0%)
High school degree	215 (35.7%)	23 (24.5%)	76 (31.5%)	116 (43.4%)
Graduate school degree	243 (40.4%)	41 (43.6%)	107 (44.4%)	95 (35.6%)
Post-graduate school degree	100 (16.6%)	21 (22.3%)	47 (19.5%)	32 (12.0%)
Weight (kg)	66.0 [21.0]	64.5 [16.3]	66.0 [22.0]	67.0 [21.0]
	69.6 ± 16.4	67.6 ± 16.8	70.3 ± 16.6	69.6 ± 16.2
Height (cm)	165.0 [11.3]	165.5 [9.5]	165.0 [13.0]	165.0 [10.0]
	166.4 ± 8.6	166.5 ± 7.6	167.2 ± 8.5	165.8 ± 8.9
BMI (kg/m2)	24.0 [6.4]	23.1 [5.6]	24.0 [6.3]	24.6 [6.7]
	25.0 ± 5.2	24.3 ± 5.6	25.1 ± 5.3	25.2 ± 4.9
**Class of BMI**				
Underweight	13 (2.2%)	2 (2.1%)	7 (2.9%)	4 (1.5%)
Normal weight	344 (57.1%)	62 (66.0%)	137 (56.8%)	145 (54.3%)
Overweight	161 (26.7%)	20 (21.3%)	64 (26.6%)	77 (28.8%)
Obesity I	61 (10.1%)	7 (7.4%)	23 (9.5%)	31 (11.6%)
Obesity II	13 (2.2%)	1 (1.1%)	5 (2.1%)	7 (2.6%)
Obesity III	10 (1.7%)	2 (2.1%)	5 (2.1%)	3 (1.1%)

Values are expressed as median and IQR in square brackets (M [IQR]) as well as mean and standard deviation (M ± SD) for continuous variables or as number and percentage (*n* (%)) for categorical variables. The Shapiro–Wilk test was performed to evaluate variables distribution. Variables are considered non-normally distributed for *p* < 0.05. BMI, body mass index.

**Table 2:** Adjusted association between respondents’ characteristics and control over-eating.

Dependent Variable	Independent Variables	Coefficient (B)	95% CI		*p*	OR
			Lower Bound	Upper Bound		
Control over-eating	Age	−0.034	0.945	0.989	**0.004**	0.967
	BMI	−0.113	0.833	0.958	**0.002**	0.893
	Dieting before COVID-19	0.830	1.375	3.822	**0.001**	2.293
	Depressed mood	−0.549	0.314	1.062	0.077	0.577
	Anxious feelings	−0.820	0.239	0.812	**0.009**	0.440
	Need to increase food intake to feel better	−1.036	0.206	0.611	**<0.001**	0.355

Multivariable binary logistic regressions between control over-eating (dependent variable) and respondents characteristics (independent co-variables). A separate univariable binary logistic regression analysis was conducted for each characteristic and the final multivariable model was determined through a backward approach. Variables included in the model: age, BMI, dieting before COVID-19, depressed mood, anxious feelings and need to increase food intake to feel better. The table shows only the final step of the regression. Statistical significance for *p* < 0.05 (in bold). BMI, body mass index; OR, odds ratio.

**Figure A1:**
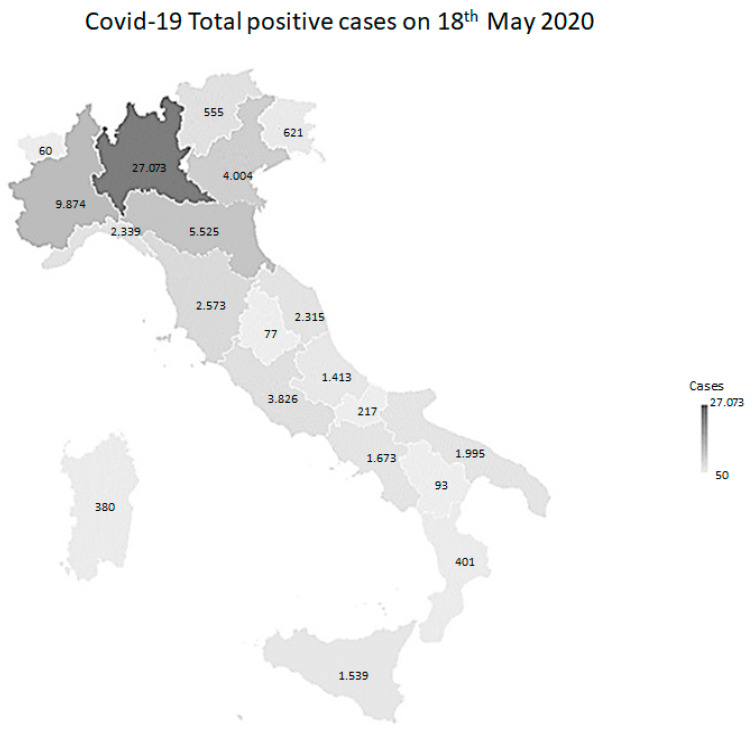
Geographical distribution of COVID-19 total positive cases in Italy on 18 May 2020. Data derived from the Health Ministry of Italy [44].

## Supplementary Materials

The following are available online at https://www.mdpi.com/2072-6643/12/7/2152/s1, Table S1: Univariable logistic regression between respondents’ characteristics and control over feeding.

## Appendix A

**Table A1:** The 28 item structured questionnaire used for the survey.

	**Questions**	**Answers**
**Personal Data**	1. Age	Age in number
	2. Gender	Female/Male/NS
	3. Place of residence	Region
	4. Hometown	Province
	5. Educational level	Elementary school diploma/Superior school diploma/Master Degree Post degree diploma
	6. Who do you live with?	Alone/With roommates/With friends/With cohabitant/With parents With children/With spouse/cohabitant and children
**Anthropometrics Data**	7. Weight	Weight in kg
	8. Height	Height in cm
**Emotional state, eating habits and emotional eating behaviors**	9. In this social isolation period, is your mood depressed?	Yes/No
	10. In this social isolation period are you focused on your work?	Yes/No/At the moment I am not working due to the pandemia
	11. In this social isolation period, are you experiencing anxious feelings?	Yes/No
	12. In this social isolation period, are you feeling “hypochondriac” (afraid of getting sick)?	Yes/No
	13. In this social isolation period, are you experiencing manifestations of anxiety (i.e., headache, sweating)?	Yes/No
	14. In this social isolation period, are you experiencing manifestations of tension, fatigability, on alert, ready to cry, trembling, restless, unable to relax?	Yes/No
	15. In this social isolation period are you experiencing breathing difficulties, choking sensation, chest pressure, dyspnea?	Yes/No
	16. In this social isolation period are you experiencing tachycardia, palpitations, chest pain, feelings of fainting?	Yes/No
	17. In this social isolation period are you taking any supplements (i.e., valerian, passionflower) and/or medications (i.e., benzodiazepines) to treat your manifestations of anxiety?	Yes/No
	18. Have you been diagnosed with medical conditions?	Yes/No
	19. In this social isolation period, are you experiencing insomnia?	Yes/No
	20. In this social isolation period, when you experience manifestations of anxiety, do you comfort yourself with foods?	Yes/No
	21. In this social isolation period, when you experience manifestations of anxiety did you avoid any food?	Yes/No
	22. Before this social isolation period, were you on a diet?	Yes/No
	23. In this social isolation period, do you continue to follow your diet?	Yes/No
	24. In this social isolation period, are you feeling guilty for your eating habits?	Yes/No
	25. In this isolation period, are you eating more to get feeling better, to reduce negative emotions or to increase pleasant feelings?	Yes/No
