# Supplementary material for: Psychological Aspects and Eating Habits during COVID-19 Home Confinement: Results of EHLC-COVID-19 Italian Online Survey
Source: Nutrients. 2020 Jul 19;12(7):2152. doi: 10.3390/nu12072152 (PMC7401000; doi:10.3390/nu12072152)
Supplement: Supplementary file 1 [file nutrients-12-02152-s001.pdf]

Table S1. Univariable binary logistic regression between respondents' characteristics and control over feeding.

	Coefficient (B)	95% CI[a]		p^a	OR
		Lower Bound	Upper Bound		
**Age**	-0.029	0.952	0.991	**0.005**	0.971
**BMI**	-0.127	0.827	0.938	**<0.001**	0.881
**Gender (ref. Males)**	-0.102	0.514	1.589	0.724	0.903
**Depressed mood**	-1.048	0.218	0.563	**<0.000**	0.350
**Anxious feelings**	-1.090	0.210	0.538	**<0.000**	0.336
**Hypochondria**	-0.044	0.602	1.520	0.852	0.957
**Dieting before COVID-19**	0.447	0.984	2.483	0.059	1.563
**Need to increase food intake to feel better**	-1.234	0.177	0.480	**<0.000**	0.291
**Insomnia**	-0.267	0.482	1.216	0.258	0.766

Univariable logistic regressions between control over feeding (dependent variable) and respondents characteristics (independent co-variables. Statistical significance for $p < 0.05$ (in bold). BMI, Body Mass Index; OR, Odd Ratio.
